# Data analysis workflow for the detection of canine vector-borne pathogens using 16 S rRNA Next-Generation Sequencing

**DOI:** 10.1186/s12917-021-02969-9

**Published:** 2021-07-31

**Authors:** Elton J. R. Vasconcelos, Chayan Roy, Joseph A. Geiger, Kristina M. Oney, Melody Koo, Songyang Ren, Brian B. Oakley, Pedro Paulo V. P. Diniz

**Affiliations:** 1 College of Veterinary Medicine, Western University of Health Sciences, 309 East 2nd Street, CA 91766 − 1854 Pomona, USA; 2 Leeds Omics, University of Leeds, LS2 9JT Leeds, United Kingdom

**Keywords:** Vector-borne diseases, vector-borne pathogens, blood microbiome, diagnostics, NGS, 16S rRNA, computational pipeline

## Abstract

**Background:**

Vector-borne diseases (VBDs) impact both human and veterinary medicine and pose special public health challenges. The main bacterial vector-borne pathogens (VBPs) of importance in veterinary medicine include *Anaplasma* spp., *Bartonella* spp., *Ehrlichia* spp., and Spotted Fever Group *Rickettsia*. Taxon-targeted PCR assays are the current gold standard for VBP diagnostics but limitations on the detection of genetically diverse organisms support a novel approach for broader detection of VBPs. We present a methodology for genetic characterization of VBPs using Next-Generation Sequencing (NGS) and computational approaches. A major advantage of NGS is the ability to detect multiple organisms present in the same clinical sample in an unsupervised (i.e. non-targeted) and semi-quantitative way. The Standard Operating Procedure (SOP) presented here combines industry-standard microbiome analysis tools with our ad-hoc bioinformatic scripts to form a complete analysis pipeline accessible to veterinary scientists and freely available for download and use at https://github.com/eltonjrv/microbiome.westernu/tree/SOP.

**Results:**

We tested and validated our SOP by mimicking single, double, and triple infections in genomic canine DNA using serial dilutions of plasmids containing the entire 16 S rRNA gene sequence of *(A) phagocytophilum, (B) v. berkhoffii*, and *E. canis*. NGS with broad-range 16 S rRNA primers followed by our bioinformatics SOP was capable of detecting these pathogens in biological replicates of different dilutions. These results illustrate the ability of NGS to detect and genetically characterize multi-infections with different amounts of pathogens in a single sample.

**Conclusions:**

Bloodborne microbiomics & metagenomics approaches may help expand the molecular diagnostic toolbox in veterinary and human medicine. In this paper, we present both *in vitro* and *in silico* detailed protocols that can be combined into a single workflow that may provide a significant improvement in VBP diagnostics and also facilitate future applications of microbiome research in veterinary medicine.

**Supplementary Information:**

The online version contains supplementary material available at 10.1186/s12917-021-02969-9.

## Background

Vector-borne diseases (VBDs) impact both human and veterinary medicine and pose special public health challenges. According to the One Health concept, animal health, environmental health, and human health are all interrelated [1]. In fact, 60 % of all human infectious diseases are classified as zoonotic diseases, having an animal origin [2]. The World Health Organization (WHO) reports that vector-borne diseases (VBD) represent more than 17 % of all known infectious diseases worldwide, causing over 700,000 annual deaths with billions of people at risk of contracting a VBD in 129 countries [3]. Data from the World Organization for Animal Health (OIE) shows that 25 % of terrestrial vertebrate pathogens of concern are vector-borne [4]. Also, the Companion Vector-borne Diseases Organization (CVBD) highlights that in more than half of the continental regions in the world, companion animals are threatened by three or more endemic VBDs [5]. Pathogen-harboring arthropods (also called vectors) such as mosquitoes, sandflies, fleas, and ticks are natural conduits for microorganisms to infect their vertebrate hosts. These complex host-pathogen relationships are constantly remodeled by environmental conditions including anthropogenic influences such as global climate change, urbanization, economic globalization, and pesticide use [6, 7]. Such phenomena increase the risk of infection of non-reservoir mammalian hosts (e.g. humans and domestic animals), which are also called incidental hosts. VBD research is therefore strategically important to maintain and improve public health.

Vector-borne pathogens (VBPs) encompass a wide variety of organisms distributed among different phylogenetic groups. In recent years, the advent and increasing adoption of Next-Generation Sequencing (NGS) and associated bioinformatics have dramatically increased our understanding of the complexity and importance of bacterial communities in a variety of environmental and clinical samples [8, 9]. For example, the Human Microbiome Project (HMP), a research consortium that started over a decade ago, has used NGS to investigate the composition and function of microbial communities in over 40 different body sites on > 30,000 samples [10]. Typically, microbiome research has been descriptive, often comparing healthy and diseased communities, considering the whole microbiota [11, 12]. As sequencing costs have continued to decline, more specific applications have become feasible such as identifying etiologic agents of disease in clinical samples for diagnostics and public health surveillance [13].

Several recent studies have characterized the microbial communities of arthropod vectors and their role in VBD transmission [14–18]. In our work, we have utilized 16 S rRNA NGS to characterize the microbiome and the presence of VBPs in cat fleas (*Ctenocephalides felis*) from Northern and Southern California [19]. Such approaches are increasingly being adopted in Veterinary Medicine, especially for the characterization of the blood microbiome of companion animals. For example, using NSG targeting the 16 S rRNA of the 18 S rRNA genes, high rates of Anaplasma platys, *Babesia vogeli, Ehrlichia canis, Hepatozoon canis*, and hemotropic *Mycoplasma* infection and co-infection were reported from a population of temple dogs in Thailand, where the NGS assay was reported to be more sensitive than conventional endpoint PCR diagnostic methods [20, 21]. Furthermore, optimizations in the amplification of bacterial DNA by using host-specific blocking primers combined with optimal DNA extraction prior to NGS further improved the sensitivity and diversity of canine VBPs in one study [22]. The use of microbiomics or metagenomics strategies for the detection and characterization of VBPs is poised to expand and benefit not only veterinary researchers but also clinicians in the near future.

NGS can be performed on any sample of interest by either direct sequencing with no PCR amplification of DNA (metagenomics) or RNA (metatranscriptomics) or targeting an amplicon, such as the 16 S rRNA gene (16 S-NGS) for prokaryotes or the 18 S rRNA gene for eukaryotes, which have become gold-standard marker genes for phylogeny and large-scale microbiome comparisons of bacterial and protozoan microorganisms. Several NGS workflows for infectious diseases already exist [13, 23], including recently published specialized methods targeting either 16 S rRNA or 18 S rRNA genes for canine VBPs comprising either bacteria or protozoa (apicomplexan piroplasms and/or euglenozoan kinetoplastids haemoparasites) [20, 21]. A need remains for a detailed step-by-step data analysis protocol for VBPs in the NGS diagnostic landscape for use by non-bioinformaticians, especially in veterinary medicine.

The goal of the current article is to describe a Standard Operating Procedures (SOP) for microbiome analyses targeting VBPs of importance in companion animals. We tested the proposed bioinformatic workflow using*in-vitro* data from selected VBPs. We focus exclusively on the 16 S-NGS approach due to its experimental affordability for diagnostics purposes and relatively straightforward computational requirements. Also, we determined the ability of NGS to detect simulated co-infections or multi-infections with VBPs. Our *in- silico* veterinary-focused microbiome methods adopt freely available industry-standard tools for 16 S-NGS analyses, adapting and executing pipelines from the QIIME [24] and Uparse [25] software packages. The computational SOP is available at https://github.com/eltonjrv/microbiome.westernu/tree/SOP and combines these existing software tools with our BASH, PERL, and R scripts to form a complete analysis pipeline accessible to veterinary scientists. To our knowledge, this is the first release of a detailed SOP for microbiome analyses applied to VBD diagnostics available free of copyright to the Veterinary Medicine community.

## Results

### 16 S-NGS Computational Workflow

**Fig. 1:**
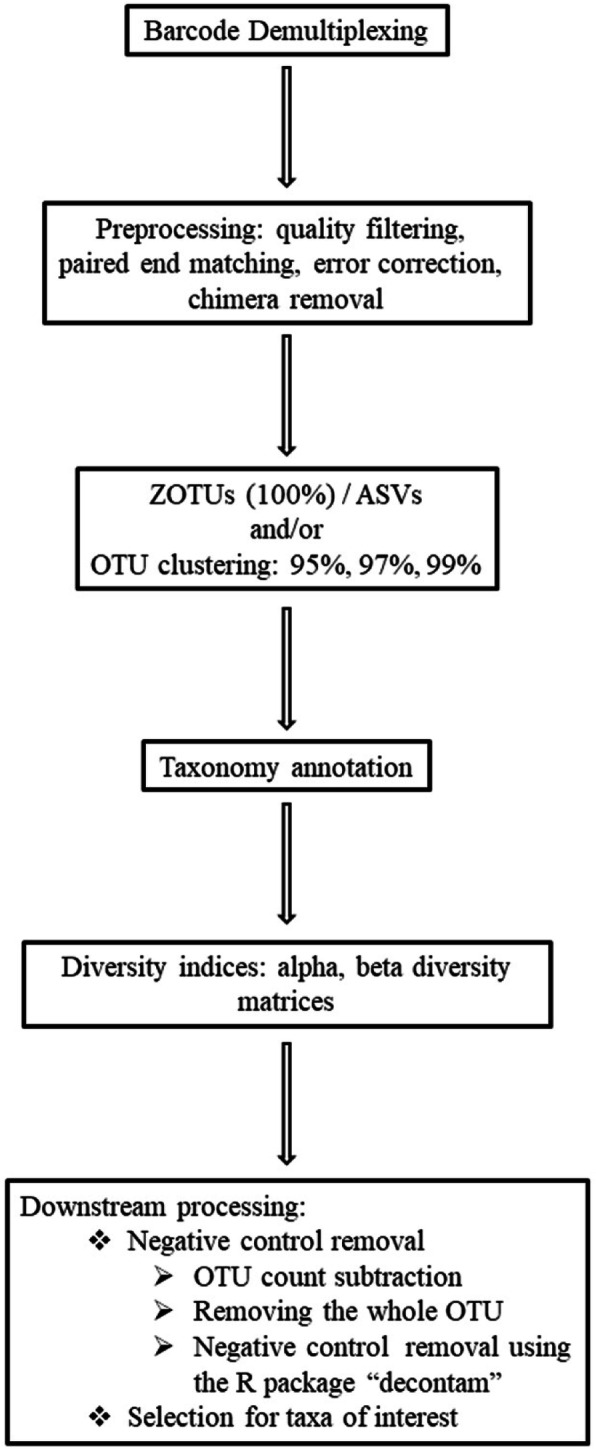
Schematic computational workflow for microbiome analysis that can be applied to VBDs diagnostics in Veterinary Medicine. See the Methods section for details

Next-generation sequencing significantly advances the field of clinical microbiology by generating millions of individual DNA sequence reads from a single sample that allows for a comprehensive evaluation of bacterial diversity that is not subject to typical limitations of culture-based approaches. A schematic microbiomics computational workflow, typically 16 S-NGS, is depicted in Fig. 1. Once followed by the *in vitro* protocol we describe in the Methods section, it can be adopted in the small animal diagnostics field, for instance.

**Fig. 2:**
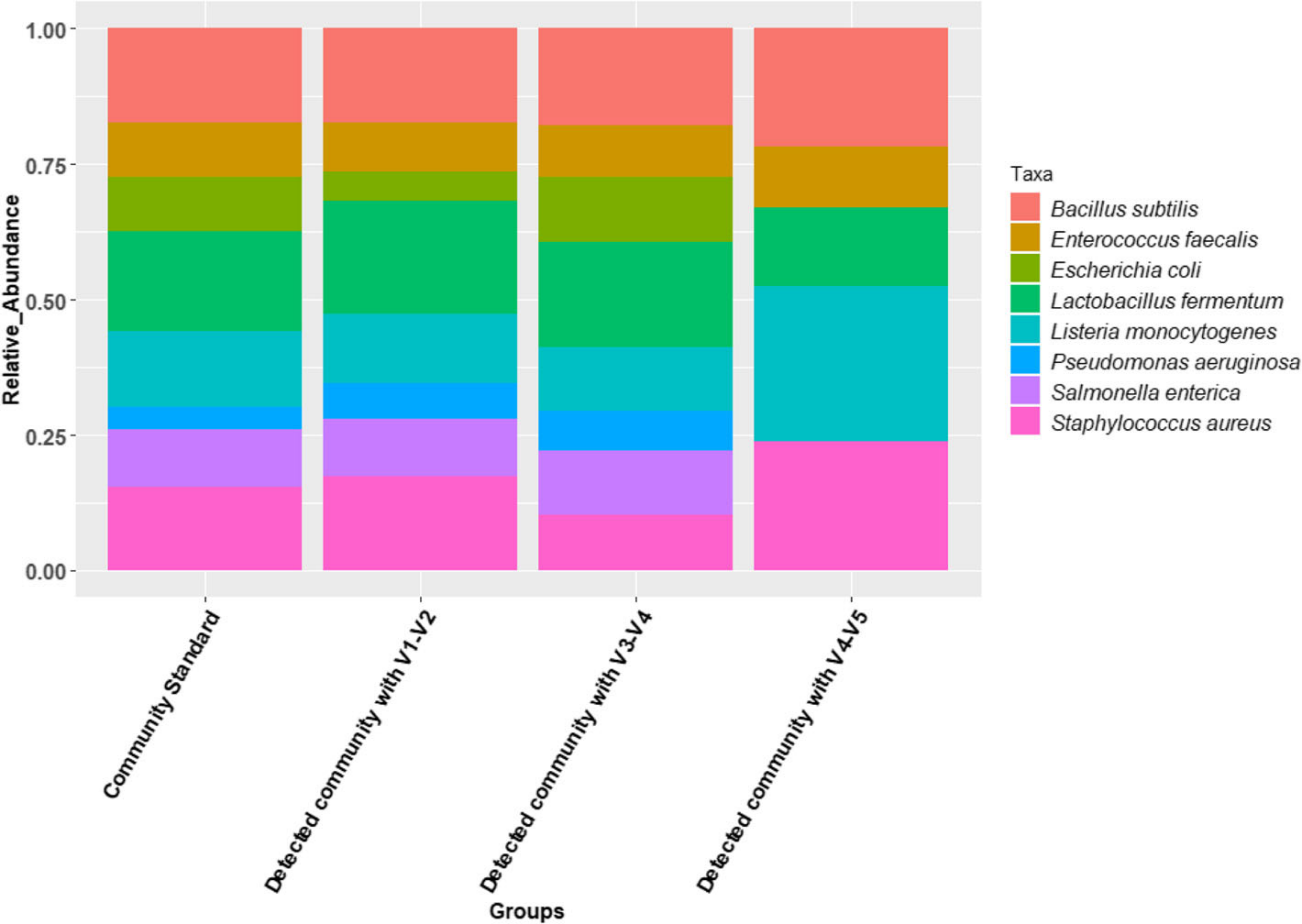
Comparison of the detection accuracy of the workflow using one of the Microbial Community Standard available with ZymoBIOMICS™. Three different variable regions; i.e. V1-V2, V3-V4, and V4-V5, of the 16 S rRNA gene were amplified in quadruplicate and analyzed. The X-axis represents the groups compared; The Y-axis represents the abundance of the microbial genera in the sample

Once sequenced, the library must pass through a bioinformatics pipeline that performs demultiplexing, quality control, error correction, operational taxonomic unit (OTU) clustering, and identification, and may include subsequent statistical hypothesis tests regarding treatment effects, etc. For the current study focused on detection and characterization of bloodborne pathogens, characterizations of the phylogenetic diversity of taxa of interest (ToI) were particularly important (Fig. 1 and SOP GitHub page disclosed herein). Comprehensive microbiome analysis typically focuses on comparing sample sets (e.g. non-infected vs. infected animals) through statistical comparisons of beta-diversity, using distance matrices (e.g. bray_curtis, jaccard) produced from the microbiome compositional data sets. Pairwise phylogenetic distances between samples can be compared using industry-standard tools like Unifrac [26] to test for significant phylogenetic differences between or among groups. Finally, a variety of data visualization approaches are commonly used such as principal coordinate analysis (PCoA) charts, along with the regular taxa relative abundance bar plots (Fig. 2).

Currently in veterinary medicine, the limited broad-range evaluation of clinical samples may restrict the detection of new and emerging zoonotic diseases. 16 S-NGS can circumvent this limitation by broad-range amplification of the whole bacterial community in a sample and can discriminate among amplicons at a single-nucleotide resolution. Phylogenetic analysis of sequence types representing putative pathogens in clinical samples is also crucial for identifying new VBD etiologic agents. Our bioinformatics SOP also provides instructions on these types of analysis and will help non-expert users to extract valuable information on potential novel pathogenic bacterial species and/or strains that might be hidden in their clinical microbiome dataset. Once generated, so-called “zero-radius operational taxonomic units” (ZOTUs), also known as “exact sequence variants” (ESVs) - which are 100 % identical 16 S amplicon sequences identified among the tens to hundreds of millions of reads from all samples (see SOP subtopic 2.2 for technical details) – can be classified by comparison against 16 S rRNA sequences from known pathogens using a phylogenetic inference approach (See SOP topic 4 for technical details). Of course, one must perform this comparison against a reliable and comprehensive 16 S database. We recommend the SILVA database [27] loaded within the ARB environment [28] for such a task.

### Detecting single and multi-infections with 16 S-NGS

The efficiency of the NGS platform involving 16 S rRNA amplicon sequencing in detecting known amounts of bacteria was verified by performing quality controls using standard microbial communities (ZymoBIOMICS™ Microbial DNA Community Standard, CA). The mock community standards consist of eight prokaryotic genera in different proportions for 16 S rRNA genes in one sample. The community standards were amplified using the primer pairs for the V1-V2, V3-V4, and V4-V5 regions of the 16 S rRNA gene and sequenced with other samples in the same MiSeq run (see Methods section). We analyzed the amplicon data for the mock community with our newly developed pipeline. We were able to successfully detect all eight prokaryotic genera from the mock community standards and the relative abundance of most of the eight genera closely matched the theoretical composition expected for the V1-V2 and V3-V4 regions. Interestingly, for the V4-V5 region, only five out of the eight genera were detected (Fig. 2).

As demonstrated by Tables 1 and 2, one of the primary advantages of NGS is its ability to detect single and multiple organisms present in the same sample. We considered a positive detection for any sample when the taxa of interest were detected at least once out of the multiple sequencing efforts. For single-infection controls, out of the six pathogens tested in six different dilutions (10^1^-10^6^), all of them were consistently detected down to the 10^2^ dilution (Table 1). Two *Bartonella* spp. and *M. haemocanis* were not detected in the 10^1^ dilutions out of the 4 trials (Table 1). For both double and triple-infections, similarly, consistent detection was obtained (Table 2). In general, 10^2^ was the minimum threshold of detection for the assays tested here (Tables 1 and 2).

**Table 1:** Summary of detection of the positive controls in the corresponding dilution for single-infection of six vector-borne pathogens. Numbers in the first bracket represent the number of times it was detected out of the number of times it was tested. Each sample was amplified for three variable regions of the 16S rRNA gene; V1-V2, V3-V4, and V4-V5

**Single Infection**						
**Vector-borne pathogen**	**10**^6^	**10**^5^	**10**^4^	**10**^3^	**10**^2^	**10**^1^
*A. phagocytophilum*	Yes(2/4)	Yes(2/4)	Yes(2/4)	Yes(2/4)	Yes(1/4)	Yes(1/4)
*B. henselae*	Yes(4/4)	Yes(4/4)	Yes(4/4)	Yes(4/4)	Yes(1/4)	No(0/4)
*B. vinsonii subsp. berkhoffii*	Yes(4/4)	Yes(4/4)	Yes(4/4)	Yes(3/4)	Yes(2/4)	No(0/4)
*E. canis*	Yes(4/4)	Yes(4/4)	Yes(4/4)	Yes(4/4)	Yes(3/4)	Yes(2/4)
*E. chaffeensis*	Yes(4/4)	Yes(4/4)	Yes(8/8)	Yes(8/8)	Yes(8/8)	Yes(1/4)
*M. haemocanis*	Yes(4/4)	Yes(4/4)	Yes(4/4)	Yes(4/4)	Yes(2/4)	No(0/4)

## Discussion

The introduction of PCR-based assays in the early 90 s led to a substantial improvement in the diagnostics of canine vector-borne diseases. However, in light of the increasing number of new tick-borne pathogens detected from dogs and humans, such as Panola Mountain *Ehrlichia* sp. [29, 30], *E. muris* [31, 32], and several new *Bartonella* spp. [33–35], VBP diagnostics may benefit from the ability of high-throughput DNA sequencing assays to detect a broader range of microbial DNA from a given sample.

Next-generation sequencing (NGS) is based on massively parallel sequencing of billions of nucleotide reads, covering the same targeted region up to 1000 times, with each read classified independently. Consequently, the identification of all sequence variants of the targeted area can be performed. NGS targeting 16 S rRNA genes was initially used to characterize environmental samples but has expanded into diagnostics in recent years [36, 37]. In two studies in ticks, NGS was able to identify the presence of novel canine and human pathogens such as *Neoehrlichia mikurensis* as well as co-infections with *Ehrlichia* and *Anaplasma* [38, 39], and *Bartonella* sp. and *Rickettsia* sp. were detected from fleas [19]. More recently, NGS was used to detect the presence of several VBPs including bacteria and protozoans, in single and co-infection, from dogs naturally infected in Thailand [20, 21], illustrating the capabilities of NGS as a molecular diagnosticmethod.

While PCR-based assays remain the current gold-standard for molecular diagnostics of vector-borne pathogens, the use of NGS is rapidly expanding due to advantages such as: detection of unknown disease-associated pathogens in clinical specimens, characterization of co-infections, investigation of microbial population diversity in the host, and strain characterization [43]. Nonetheless, there are several barriers to the broader use of NGS in molecular diagnostics, including but not limited to: carryover microbial DNA contaminants from sample collection tools and lab reagents, sequencing error rates, elevated cost, result turnover time, analytical sensitivity, and preferential amplification of dominant microbial sequences [40, 41]. As demonstrated in Fig. 2, different sets of primers targeting distinct variable regions of the 16 S rRNA gene may yield different results regarding the relative abundance of microbial communities. Also, the analytical sensitivity and limit of detection (LOD) achieved by the 16 S NGS assays performed in this study remain sub-optimal when compared to the LOD reached by our conventional or real-time PCR assays for some of these pathogens [42, 43]. Other research groups have reported NGS sensitivity to canine VBPs comparable or even superior to conventional endpoint PCR [20, 21] The targeting of the 16 S rRNA gene has also limited capability in defining species within genera where this region is highly conserved, such as *Rickettsia* spp. and *Brucella* spp. Future advances in NGS technology such as longer read lengths are expected to address some of these limitations, making it a potentially transformative tool for the diagnosis of new or emerging pathogens in animals and humans.

In this methodology article, we disclosed both benchtop protocols and a “step-by-step” bioinformatics tutorial for performing microbiome assays and analyses, respectively, on VBD diagnostics in Veterinary Medicine. While in this article we only report the results from synthetic positive controls, we have successfully used this bioinformatic pipeline to detect the presence of *Anaplasma phagocytophilum* or *Ehrlichia ewingii* in dog blood [44].

## Conclusions

The detailed 16 S-NGS protocols for microbiome analyses provided herein can efficiently and accurately characterize mock communities and may also be able to detect co- and multi-infections of VBPs. These procedures can be combined into a single workflow that should facilitate future applications of microbiome approaches to VBD diagnostics in Veterinary Medicine. Such approaches may improve current understandings of VBDs and their impact on both Veterinary and Human Medicine.

## Methods

### Positive controls and standard curves

Synthesis of positive controls and generation of standard curves were completed by cloning full 16S rRNA sequences of *Anaplasma phagocytophilum*, *Ehrlichia canis, E. chaffeensis, B. henselae, Bartonella vinsonii berkhoffii (B.v.b)*, and *Mycoplasma haemocanis* into plasmids (Eurofins Genomics LLC, Louisville, KY, USA). *A*. *phagocytophilum* positive control was generated from the consensus sequence derived after careful analysis of the 16S rRNA sequence alignment associated with the following accession numbers; CP006618, CP006616, APHH01000002, NC_007797, and CP006617. Similarly, for *E. canis*, a consensus sequence of strains Jake (CP000107), Oklahoma (NR_118741), Florida (M73226), and Malaysia (KR920044) was synthesized. For *E. chaffeensis*, strain Arkansas (NC_007799) was used. For *B. henselae* strain Houston-1 (CP020742) was used as a positive control whereas for B. *vinsonii berkhoffii* Winnie (CP003124) was used. Lastly *M. haemocanis* Illinois (CP003199) was used as a reference to generate the positive control. Standardized ten-fold dilutions were then generated for each plasmid from 1 × 10^7^ to 0.1 copies per microliter (µL). The dilutions were made with dog gDNA to simulate natural infection. The gDNA concentration from this dog was quantified by spectrophotometry (average concentration of 27.8 ± 1.1 ng/dL with average 260/280 ratio of 1.88 ± 0.05) and the absence of PCR inhibitors was demonstrated by amplification of a fragment of the glyceraldehyde-3-phosphate dehydrogenase gene, as described previously [45]. Positive samples were diluted using VBP free EDTA-whole blood, confirmed using the molecular vector-borne disease panel from Vector-Borne Disease Diagnostic Laboratory at North Carolina State University College of Veterinary Medicine which tests for the following genera: *Anaplasma, Babesia, Bartonella, Ehrlichia*, Hemotropic *Mycoplasma*, Spotted-Fever *Rickettsia*, and *Leishmania* [46–49]. A different modified forward primer AE16S_45F (5’ AGCYTAACACATGCAAGTCGAACG3’) was used to detect *Anaplasma* and *Ehrlichia* [48] in the real-time PCR assay.

### NGS library preparation and sequencing

For 16 S rRNA gene regions V1-V2 (~ 350 bp) and V3-V4 (~ 460 bp), the NGS libraries were created with proprietary primers from Zymo (Zymo Quick-16 S NGS Library Prep Kit, Zymo Research, Irvine, CA). For the V4-V5 region, universal primers (~ 550 bp) were used as previously described [50], followed by a barcoding scheme based on Faircloth and Glenn [51] as previously used by our research group [52]. These barcoding tags have been validated with the EDITTAG algorithm as previously described [51] and shown to be less error-prone than earlier barcode designs [53]. Illumina MiSeq 2 × 300 bp v2 kit was used to sequence the library at the University of Southern California Genome Core Center.

### Library preparation quality control

During library preparation, utmost importance was given to quality control. A biosafety cabinet was used for sample preparation in separate and dedicated space. As described in detail in Oney et al. (44) adequate measures were taken to prevent barcode cross-contamination. Libraries were further purified by magnetic clean-up, were quantified by automated electrophoresis (BioAnalyzer 2100, Agilent, Santa Clara, CA), and pooled according to Illumina recommendations [54].

### Simulation of co-infection with synthetic plasmids

For single infections all total six different pathogens were amplified for three different 16 S rRNA variable regions; V1-V2, V3-V4, & V4-V5. Among them *(A) phagocytophilum, (B) henselae, B. vinsonii subsp. berkhoffii, E. canis, and M. haemocanis* were tested in quadruplicate, whereas *E. chaffeensis* was tested eight times. Serial dilutions 1 × 10^6^ through 1 × 10^1^ copies/µL for all six synthetic plasmids were created. To simulate different possibilities of co-infection, *A. phagocytophilum* serial dilutions of 1 × 10^6^ to 1 × 10^2^ were mixed in descending order of copies per reaction with *E. canis* which was at a fixed concentration of 1 × 10^6^ for all reactions. In another effort, *(A) phagocytophilum* serial dilutions of 1 × 10^4^ to 1 × 10^2^ were mixed with *(B) henselae* at a fixed concentration of 1 × 10^4^. These conditions were also used to simulate triple co-infection consisting of *(A) phagocytophilum, E. canis*, and *(B) vinsonii subsp. berkhoffii* with *E. canis* being the standard at 1 × 10^6^ copies per reaction while *(A) phagocytophilum* and *(B) vinsonii subsp. berkhoffii* were mixed in decreasing order of concentration of 1 × 10^6^ to 1 × 10^2^. Similarly, for another triple infection control fixed dilutions (10^4^) of *E. canis* and *M. haemocanis* were mixed with different dilutions (1 × 10^4^ to 1 × 10^2^) of *B. vinsonii subsp. berkhoffii*. All the double and triple infections were also repeated quadruplicate times or more (Tables 1 and 2).

**Table 2:** Summary of detection of the positive controls in the corresponding dilution for co-infections of six vector-borne pathogens. Numbers in the first bracket represent the number of times it was detected out of the number of times it was tested. Each sample was amplified for three variable regions of the 16S rRNA gene; V1-V2, V3-V4, and V4-V5

**Double Infection**					
***A. phagocytophilum * plus *E. canis***					
**Vector-borne pathogen**	**10**^6^**:10**^6^	**10**^5^**:10**^6^	**10**^4^**:10**^6^	**10**^3^**:10**^6^	**10**^2^**:10**^6^
*A. phagocytophilum*	Yes(4/4)	Yes(4/4)	Yes(4/4)	Yes(2/4)	No(0/4)
*E. canis*	Yes(4/4)	Yes(4/4)	Yes(4/4)	Yes(4/4)	Yes(4/4)
***A. phagocytophilum*plus* B. henselae***					
**Vector-borne pathogen**	**10**^4^**:10**^4^	**10**^3^**:10**^4^	**10**^2^**:10**^4^		
*A. phagocytophilum*	Yes(4/4)	Yes(4/4)	Yes(4/4)		
*B. henselae*	Yes(4/4)	Yes(4/4)	Yes(4/4)		
**Triple infection**					
***A. phagocytophilum*plus *E. canis *plus *B. vinsonii subsp. berkhoffii***					
Vector-borne pathogen	**10**^6^**:10**^6^**:10**^6^	**10**^5^**:10**^6^**:10**^5^	**10**^4^**:10**^6^**:10**^4^	**10**^3^**:10**^6^**:10**^3^	**10**^2^**:10**^6^**:10**^2^
*A. phagocytophilum*	**Yes(6/6)**	**Yes(6/6)**	**Yes(6/6)**	**Yes(3/6)**	**No(0/6)**
*E. canis*	**Yes(6/6)**	**Yes(6/6)**	**Yes(6/6)**	**Yes(6/6)**	**Yes(6/6)**
*B. vinsonii subsp. berkhoffii *	**Yes(3/6)**	**Yes(3/6)**	**Yes(3/6)**	**Yes(1/6)**	**Yes(1/6)**
***E. canis *plus* B. vinsonii subsp. berkhoffii *plus* M. haemocanis***					
Vector-borne pathogen	**10**^4^**:10 **^4^**:10 **^4^	**10**^4^**:10**^3^**:10**^4^	**10**^4^**:10**^2^**:10**^4^		
*E. canis*	**Yes(4/4)**	**Yes(4/4)**	**Yes(4/4)**		
*B. vinsonii subsp. berkhoffii*	**Yes(4/4)**	**Yes(4/4)**	**Yes(1/4)**		
*M. haemocanis*	**Yes(4/4)**	**Yes(4/4)**	**Yes(4/4)**		

### PCR amplification, barcoding, and amplicon sequencing

Custom dual indexed adapters were used to amplify the single, double and triple co-infections targeting the V1-V2 (~ 349 bp), V3-V4 (~ 460 bp), and V4-V5 (~ 410 bp) regions of the 16 S rRNA gene. The positive control was the ZymoBIOMICS™ Microbial Community DNA Standard (Zymo Research, Irvine, CA). Amplification for the first PCR reaction was performed by conventional PCR using the Eppendorf Master cycler using single hot start at 94 °C for 2 min, futher 30 cycles of denaturing at 94 °C, annealing at 57 °C for 45 s, and final extension at 72 °C for 1 min, with the PCR reaction in a 25 µL final volume containing 10X Ex Taq Buffer, dNTPs, and Takara Ex Taq HS (Takara Bio Inc., Shiga, Japan), 10 pmol of each primer, 2 µL of DNA template. The second amplification of 15 cycles was performed with the same conditions as the first, but with 1 µL of template coming from the primary inoculation. With 16 S rRNA primers, the target amplicon was amplified during the first step. In the second step, i5 and i7 barcoded primers in 96-well configurations were used to form a library for sequencing [51]. Negative controls consisted of molecular-grade water and gDNA from an uninfected dog used to prepare the serial dilutions of positive controls, to characterize any bacterial DNA contaminants from the PCR reagents, as well as from the gDNA used, respectively. 1.5 % agarose gel electrophoresis with 1kp Plus DNA Ladder. V4-V5 amplicons were then normalized according to the manufacturer’s instructions (SequalPrep Normalization Plate kit, ThermoFisher Scientific, Waltham, MA, USA) and were then pooled into 5mL tubes and stored at -30 °C. V1-V2 and V3-V4 amplicons were normalized using the ZymoBIOMICS™ 96 MagBead DNA Kit (Zymo Research, Irvine, CA, USA) as per the manufacturer’s protocol. Samples were sequenced with Illumina MiSeq paired-end V3 sequencing chemistry, with three to four 96-well plates simultaneously multiplexed per run. The number of raw and processed reads is available in Supplementary Table 1.

#### Bioinformatics pipeline on Mock samples analysis

Our computational pipeline for 16 S-NGS microbiome analysis is available in detail as a Standard Operating Procedure (SOP) publicly available at https://github.com/eltonjrv/microbiome.westernu/tree/SOP. Besides detailed ordered steps and useful commented commands, the SOP URL above also provides accessible links for in-house supporting scripts plus a customized RDP reference database [55] for a better taxonomic classification of bacterial VBPs of interest in Veterinary Medicine (https://github.com/eltonjrv/microbiome.westernu/tree/refDB). Due to the open-source nature of most of the adopted programs, the SOP must be run on a Unix Platform (either Mac or Linux). After installing the required tools, both Unix-familiar users and novices will be able to run it without any further difficulties, since we have provided detailed explanations for each step of the pipeline.

In brief, the sequence data were initially checked for barcodes with QIIME v1.9 extract_barcodes.py script [55], then demultiplexed with the new “qiime demux emp-paired” function from the QIIME2 package [56]. Primers were removed using Trimmomatic [55]. Uparse tool was applied using the new -unoise3 function for modeling and correcting Illumina-sequenced amplicon errors, including detection and removal of chimeras, as well as generating operational taxonomic unit (OTU) tables at a 100 % identity level, also called as ZOTUs (zero-radius OTUs) or ESVs (Exact Sequence Variant) (please refer to our *ad hoc* Uparse bash script available at https://github.com/eltonjrv/microbiome.westernu/blob/master/run-uparse-amp250-450-ZOTUs.bash). In addition to the ZOTUs, we have also provided an advantageous feature from Usearch regarding OTU clustering at 95 %, 97 %, and 99 % identity levels for comparison purposes (https://github.com/eltonjrv/microbiome.westernu/blob/master/run-uparse-amp250-450-OTUs95_97_99_100.bash). Users may want to have the flexibility to work downstream with any relevant percentage of identity threshold that suits their data.

Background abundance of microbial taxa is a major hindrance for accurate analysis of low diversity samples. The background diversity is expected to be present in every sample and our previous data and other studies have shown that the frequency of contamination is directly related to the sample DNA abundance within total DNA content [44, 57, 58]. Along with the existing tools to remove such contamination from the sample [57–59], we have provided two new options in our pipeline to deal with such background contaminations in the dataset: (1) An ad-hoc R script was written for subtracting negative control-derived ZOTU counts from target samples (see SOP step 3.2.1). The use of blank negative control(s) with no sample DNA is of utmost importance for the analysis of low diversity samples. Users shall analyze the blank sets as negative-control samples (with no externally supplied DNA) and generate ZOTUs/OTUs using the same parameters as individual samples. Afterward, using the first utility users can remove the abundance present in the negative control from the actual samples at ZOTU/OTU level. In addition to removing the background diversity, this particular option has its usefulness in dealing with cross-contamination as well. In the latter case, if cross-contamination occurs during the sample preparation step from the actual sample to the blank negative control, during negative control removal it will not remove the ZOTU/OTU completely from the sample. The abundance of contaminant ZOTU/OTU in the blank control set theoretically cannot surpass the abundance of that particular ZOTU/OTU in the actual sample. (2) An alternative *ad hoc* PERL code is provided for removing the whole ZOTUs/OTUs content present in negative controls from the actual samples to be assessed. Through that PERL code, users can remove the whole negative control-derived ZOTUs/OTUs content from their target samples, rather than count subtraction (see SOP steps 3.2.2 and 3.2.3). After generating ZOTUs/OTUs from the blank controls like earlier, this option will match every ZOTU/OTU present in the control to the sample ZOTUs/OTUs and remove them from the latter. Though this option can be equally effective while dealing with contaminations from background negative controls to the samples, users need to be cautious for the cross-contamination that may happen during the sample preparation step from actual samples to the negative controls.

## Supplementary information

**Additional file 1**.

## Data Availability

All raw sequencing data from mock samples generated and analyzed in this study are available in the NCBI Sequence Read Archive (SRA) repository, under the following accession number: PRJNA667928.

## Abbreviations

rRNA: ribosomal ribonucleic acid
VBP: Vector-borne pathogen
VBD: Vector-borne disease
NGS: Next-generation sequencing
SOP: Standard Operating Procedure
***A. phagocytophilum***: *Anaplasma phagocytophilum*
B. henselae: *Bartonella henselae*
B. v. berkhoffii: *Bartonella vinsonii* subsp. *berkhoffii*
E. canis: *Ehrlichia canis*
E. chaffeensis: *Ehrlichia chaffeensis*
E. muris: *Ehrlichia muris*
M. haemocanis: *Mycoplasma haemocanis*
WHO: World Health Organization
OIE: World Organization for Animal health
HMP: Human Microbiome Project
DNA: Deoxyribonucleic acid
RNA: Ribonucleic acid
OTU: Operational taxonomic unit
ToI: Taxa of interest
PCoA: Principal coordinate analysis
ZOTU: Zero-radius operational taxonomic unit
ESV: Exact sequence variant
PCR: Polymerase chain reaction
LOD: Limit of detection
EDTA: Ethylenediamine tetra-acetic acid
URL: Uniform resource locator

## References

[1] King LJ, Anderson LR, Blackmore CG, Blackwell MJ, Lautner EA, Marcus LC, Meyer TE, Monath TP, Nave JE, Ohle J (2008). Executive summary of the AVMA One Health Initiative Task Force report. J Am Vet Med Assoc.

[2] Torrey EF, Yolken RH. Beasts of the Earth: Animals, Humans, and Disease. New Brunswick: Rutgers University Press; 2005.

[3] Vector-borne Diseases http://www.who.int/news-room/fact-sheets/detail/vector-borne-diseases.

[4] Stuchin M, Machalaba CC, Karesh WB. Vector-borne Diseases: Animals and Patterns. In: Global Health Impacts of Vector-Borne Diseases: Workshop Summary. edn. Washington (DC): National Academies Press; 2016. 27054234

[5] Occurrences map of CVBDs [http://www.cvbd.org/en/occurrence-maps/world-map/].

[6] Rio RVM, Attardo GM, Weiss BL (2016). Grandeur Alliances: Symbiont Metabolic Integration and Obligate Arthropod Hematophagy. Trends Parasitol.

[7] Shaw WR, Catteruccia F. Vector biology meets disease control: using basic research to fight vector-borne diseases. Nat Microbiol. 2019;4(1):20-34. 10.1038/s41564-018-0214-7 PMC6437764 30150735

[8] Knight R, Vrbanac A, Taylor BC, Aksenov A, Callewaert C, Debelius J, Gonzalez A, Kosciolek T, McCall LI, McDonald D (2018). Best practices for analysing microbiomes. Nat Rev Microbiol.

[9] Mallick H, Ma S, Franzosa EA, Vatanen T, Morgan XC, Huttenhower C (2017). Experimental design and quantitative analysis of microbial community multiomics. Genome Biol.

[10] Human Microbiome Project Data Portal [https://portal.hmpdacc.org/].

[11] Langille MG, Zaneveld J, Caporaso JG, McDonald D, Knights D, Reyes JA, Clemente JC, Burkepile DE, Vega Thurber RL, Knight R (2013). Predictive functional profiling of microbial communities using 16S rRNA marker gene sequences. Nat Biotechnol.

[12] Lloyd-Price J, Mahurkar A, Rahnavard G, Crabtree J, Orvis J, Hall AB, Brady A, Creasy HH, McCracken C, Giglio MG (2017). Strains, functions and dynamics in the expanded Human Microbiome Project. Nature.

[13] Afshinnekoo E, Chou C, Alexander N, Ahsanuddin S, Schuetz AN, Mason CE (2017). Precision Metagenomics: Rapid Metagenomic Analyses for Infectious Disease Diagnostics and Public Health Surveillance. J Biomol Tech.

[14] Abraham NM, Liu L, Jutras BL, Yadav AK, Narasimhan S, Gopalakrishnan V, Ansari JM, Jefferson KK, Cava F, Jacobs-Wagner C (2017). Pathogen-mediated manipulation of arthropod microbiota to promote infection. Proc Natl Acad Sci U S A.

[15] Bonnet SI, Binetruy F, Hernandez-Jarguin AM, Duron O (2017). The Tick Microbiome: Why Non-pathogenic Microorganisms Matter in Tick Biology and Pathogen Transmission. Front Cell Infect Microbiol.

[16] Couper L, Swei A. Tick microbiome characterization by Next-Generation 16S rRNA amplicon sequencing. J Vis Exp. 2018;(138):58239. 10.3791/58239 PMC6231894 30199026

[17] Finney CA, Kamhawi S, Wasmuth JD (2015). Does the arthropod microbiota impact the establishment of vector-borne diseases in mammalian hosts?. PLoS Pathog.

[18] Kelly PH, Bahr SM, Serafim TD, Ajami NJ, Petrosino JF, Meneses C, Kirby JR, Valenzuela JG, Kamhawi S, Wilson ME. The Gut Microbiome of the Vector Lutzomyia longipalpis Is Essential for Survival of Leishmania infantum. MBio. 2017;8(1):e01121-16. 10.1128/mBio.01121-16 PMC5241394 28096483

[19] Vasconcelos EJR, Billeter SA, Jett LA, Meinersmann RJ, Barr MC, Diniz P, Oakley BB. Assessing Cat Flea Microbiomes in Northern and Southern California by 16S rRNA Next-Generation Sequencing. Vector Borne Zoonotic Dis. 2018;18(9):491-9. 10.1089/vbz.2018.2282 29893631

[20] Huggins LG, Koehler AV, Ng-Nguyen D, Wilcox S, Schunack B, Inpankaew T, Traub RJ (2019). A novel metabarcoding diagnostic tool to explore protozoan haemoparasite diversity in mammals: a proof-of-concept study using canines from the tropics. Sci Rep.

[21] Huggins LG, Koehler AV, Ng-Nguyen D, Wilcox S, Schunack B, Inpankaew T, Traub RJ (2019). Assessment of a metabarcoding approach for the characterisation of vector-borne bacteria in canines from Bangkok, Thailand. Parasit Vectors.

[22] Huggins LG, Koehler AV, Schunack B, Inpankaew T, Traub RJ. A Host-Specific Blocking Primer Combined with Optimal DNA Extraction Improves the Detection Capability of a Metabarcoding Protocol for Canine Vector-Borne Bacteria. Pathogens. 2020;9(4):258. 10.3390/pathogens9040258 PMC7238069 32244645

[23] Whittle E, Leonard MO, Harrison R, Gant TW, Tonge DP (2018). Multi-Method Characterization of the Human Circulating Microbiome. Front Microbiol.

[24] Caporaso JG, Kuczynski J, Stombaugh J, Bittinger K, Bushman FD, Costello EK, Fierer N, Pena AG, Goodrich JK, Gordon JI (2010). QIIME allows analysis of high-throughput community sequencing data. Nat Methods.

[25] Edgar RC (2013). UPARSE: highly accurate OTU sequences from microbial amplicon reads. Nat Methods.

[26] Lozupone C, Knight R (2005). UniFrac: a new phylogenetic method for comparing microbial communities. Appl Environ Microbiol.

[27] Yilmaz P, Parfrey LW, Yarza P, Gerken J, Pruesse E, Quast C, Schweer T, Peplies J, Ludwig W, Glockner FO (2014). The SILVA and “All-species Living Tree Project (LTP)” taxonomic frameworks. Nucleic Acids Res.

[28] Ludwig W, Strunk O, Westram R, Richter L, Meier H, Yadhukumar, Buchner A, Lai T, Steppi S, Jobb G (2004). ARB: a software environment for sequence data. Nucleic Acids Res.

[29] Qurollo BA, Davenport AC, Sherbert BM, Grindem CB, Birkenheuer AJ, Breitschwerdt EB (2013). Infection with Panola Mountain Ehrlichia sp. in a dog with atypical lymphocytes and clonal T-cell expansion. J Vet Intern Med.

[30] Reeves WK, Loftis AD, Nicholson WL, Czarkowski AG (2008). The first report of human illness associated with the Panola Mountain Ehrlichia species: a case report. J Med Case Rep.

[31] Hegarty BC, Maggi RG, Koskinen P, Beall MJ, Eberts M, Chandrashekar R, Breitschwerdt EB (2012). Ehrlichia muris infection in a dog from Minnesota. J Vet Intern Med.

[32] Johnson DK, Schiffman EK, Davis JP, Neitzel DF, Sloan LM, Nicholson WL, Fritsche TR, Steward CR, Ray JA, Miller TK (2015). Human Infection with Ehrlichia muris-like Pathogen, United States, 2007–2013(1). Emerg Infect Dis.

[33] Chomel BB, McMillan-Cole AC, Kasten RW, Stuckey MJ, Sato S, Maruyama S, Diniz PP, Breitschwerdt EB (2012). Candidatus Bartonella merieuxii, a potential new zoonotic Bartonella species in canids from Iraq. PLoS Negl Trop Dis.

[34] Diniz PP, Billeter SA, Otranto D, De Caprariis D, Petanides T, Mylonakis ME, Koutinas AF, Breitschwerdt EB (2009). Molecular documentation of Bartonella infection in dogs in Greece and Italy. J Clin Microbiol.

[35] Eremeeva ME, Gerns HL, Lydy SL, Goo JS, Ryan ET, Mathew SS, Ferraro MJ, Holden JM, Nicholson WL, Dasch GA (2007). Bacteremia, fever, and splenomegaly caused by a newly recognized bartonella species. N Engl J Med.

[36] Dunne WM, Westblade LF, Ford B (2012). Next-generation and whole-genome sequencing in the diagnostic clinical microbiology laboratory. Eur J Clin Microbiol Infect Dis.

[37] Salipante SJ, Sengupta DJ, Rosenthal C, Costa G, Spangler J, Sims EH, Jacobs MA, Miller SI, Hoogestraat DR, Cookson BT (2013). Rapid 16S rRNA next-generation sequencing of polymicrobial clinical samples for diagnosis of complex bacterial infections. PLoS One.

[38] Gofton AW, Doggett S, Ratchford A, Oskam CL, Paparini A, Ryan U, Irwin P (2016). Bacterial Profiling Reveals Novel “*Candidatus* Neoehrlichia”, *Ehrlichia*, and *Anaplasma* Species in Australian Human-Biting Ticks. PLoS ONE.

[39] Trout Fryxell RT, DeBruyn JM. The microbiome of *Ehrlichia*-infected and uninfected lone star ticks** (***Amblyomma americanum*). PLoS One. 2016;11(1):e0146651. 10.1371/journal.pone.0146651 PMC4709196 26751816

[40] Simner PJ, Miller S, Carroll KC (2018). Understanding the Promises and Hurdles of Metagenomic Next-Generation Sequencing as a Diagnostic Tool for Infectious Diseases. Clin Infect Dis.

[41] Miller S, Chiu C, Rodino KG, Miller MB. Point-counterpoint: should we be performing metagenomic next-generation sequencing for infectious disease diagnosis in the clinical laboratory? J Clin Microbiol. 2020;58(3):e01739-19. 10.1128/JCM.01739-19 PMC7041574 31619533

[42] Chan D, Geiger JA, Vasconcelos EJR, Oakley B, Diniz P (2018). Bartonella rochalimae Detection by a Sensitive and Specific PCR Platform. Am J Trop Med Hyg.

[43] Diniz PP, Morton BA, Tngrian M, Kachani M, Barron EA, Gavidia CM, Gilman RH, Angulo NP, Brenner EC, Lerner R (2013). Infection of domestic dogs in peru by zoonotic bartonella species: a cross-sectional prevalence study of 219 asymptomatic dogs. PLoS Negl Trop Dis.

[44] Oney K, Koo M, Roy C, Ren S, Qurollo B, Juhasz NB, Vasconcelos EJR, Oakley B, Diniz P. Evaluation of a commercial microbial enrichment kit used prior DNA extraction to improve the molecular detection of vector-borne pathogens from naturally infected dogs. J Microbiol Methods. 2021;10:106163. 10.1016/j.mimet.2021.106163 33581169

[45] Birkenheuer AJ, Levy MG, Breitschwerdt EB (2003). Development and evaluation of a seminested PCR for detection and differentiation of Babesia gibsoni (Asian genotype) and B. canis DNA in canine blood samples. J Clin Microbiol.

[46] Hegarty BC, Qurollo BA, Thomas B, Park K, Chandrashekar R, Beall MJ, Thatcher B, Breitschwerdt EB (2015). Serological and molecular analysis of feline vector-borne anaplasmosis and ehrlichiosis using species-specific peptides and PCR. Parasit Vectors.

[47] Tyrrell JD, Qurollo BA, Tornquist SJ, Schlaich KG, Kelsey J, Chandrashekar R, Breitschwerdt EB (2019). Molecular identification of vector-borne organisms in Ehrlichia seropositive Nicaraguan horses and first report of Rickettsia felis infection in the horse. Acta Trop.

[48] von Fricken ME, Qurollo BA, Boldbaatar B, Wang YW, Jiang RR, Lkhagvatseren S, Koehler JW, Moore TC, Nymadawa P, Anderson BD (2020). Genetic diversity of Anaplasma and Ehrlichia bacteria found in Dermacentor and Ixodes ticks in Mongolia. Ticks Tick Borne Dis.

[49] Qurollo BA, Archer NR, Schreeg ME, Marr HS, Birkenheuer AJ, Haney KN, Thomas BS, Breitschwerdt EB (2017). Improved molecular detection of Babesia infections in animals using a novel quantitative real-time PCR diagnostic assay targeting mitochondrial DNA. Parasit Vectors.

[50] Wong K, Shaw TI, Oladeinde A, Glenn TC, Oakley B, Molina M (2016). Rapid Microbiome Changes in Freshly Deposited Cow Feces under Field Conditions. Front Microbiol.

[51] Faircloth BC, Glenn TC (2012). Not all sequence tags are created equal: designing and validating sequence identification tags robust to indels. PLoS One.

[52] Oakley BB, Lillehoj HS, Kogut MH, Kim WK, Maurer JJ, Pedroso A, Lee MD, Collett SR, Johnson TJ, Cox NA (2014). The chicken gastrointestinal microbiome. FEMS Microbiol Lett.

[53] Hamady M, Walker JJ, Harris JK, Gold NJ, Knight R (2008). Error-correcting barcoded primers for pyrosequencing hundreds of samples in multiplex. Nat Methods.

[54] 16S Metagenomic Sequencing Library Preparation: Preparing 16S Ribosomal RNA Gene Amplicons for the Illumina MiSeq System https://support.illumina.com/documents/documentation/chemistry_documentation/16s/16s-metagenomic-library-prep-guide-15044223-b.pdf.

[55] Cole JR, Wang Q, Fish JA, Chai B, McGarrell DM, Sun Y, Brown CT, Porras-Alfaro A, Kuske CR, Tiedje JM (2014). Ribosomal Database Project: data and tools for high throughput rRNA analysis. Nucleic Acids Res.

[56] Bolyen E, Rideout JR, Dillon MR, Bokulich NA, Abnet CC, Al-Ghalith GA, Alexander H, Alm EJ, Arumugam M, Asnicar F, Bai Y, Bisanz JE, Bittinger K, Brejnrod A, Brislawn CJ, Brown CT, Callahan BJ, Caraballo-Rodríguez AM, Chase J, Cope EK, Da Silva R, Diener C, Dorrestein PC, Douglas GM, Durall DM, Duvallet C, Edwardson CF, Ernst M, Estaki M, Fouquier J, Gauglitz JM, Gibbons SM, Gibson DL, Gonzalez A, Gorlick K, Guo J, Hillmann B, Holmes S, Holste H, Huttenhower C, Huttley GA, Janssen S, Jarmusch AK, Jiang L, Kaehler BD, Kang KB, Keefe CR, Keim P, Kelley ST, Knights D, Koester I, Kosciolek T, Kreps J, Langille MGI, Lee J, Ley R, Liu YX, Loftfield E, Lozupone C, Maher M, Marotz C, Martin BD, McDonald D, McIver LJ, Melnik AV, Metcalf JL, Morgan SC, Morton JT, Naimey AT, Navas-Molina JA, Nothias LF, Orchanian SB, Pearson T, Peoples SL, Petras D, Preuss ML, Pruesse E, Rasmussen LB, Rivers A, Robeson MS, Rosenthal P, Segata N, Shaffer M, Shiffer A, Sinha R, Song SJ, Spear JR, Swafford AD, Thompson LR, Torres PJ, Trinh P, Tripathi A, Turnbaugh PJ, Ul-Hasan S, van der Hooft JJJ, Vargas F, Vázquez-Baeza Y, Vogtmann E, von Hippel M, Walters W, Wan Y, Wang M, Warren J, Weber KC, Williamson CHD, Willis AD, Xu ZZ, Zaneveld JR, Zhang Y, Zhu Q, Knight R, and Caporaso JG. Reproducible, interactive, scalable and extensible microbiome data science using QIIME 2. Nature Biotechnology. 2019;37:852–7.

[57] Davis NM, Proctor DM, Holmes SP, Relman DA, Callahan BJ (2018). Simple statistical identification and removal of contaminant sequences in marker-gene and metagenomics data. Microbiome.

[58] Jervis-Bardy J, Leong LE, Marri S, Smith RJ, Choo JM, Smith-Vaughan HC, Nosworthy E, Morris PS, O’Leary S, Rogers GB (2015). Deriving accurate microbiota profiles from human samples with low bacterial content through post-sequencing processing of Illumina MiSeq data. Microbiome..

[59] Karstens L, Asquith M, Davin S, Fair D, Gregory WT, Wolfe AJ, Braun J, McWeeney S. Controlling for Contaminants in Low-Biomass 16S rRNA Gene Sequencing Experiments. mSystems. 2019;4(4):e00290-19. 10.1128/mSystems.00290-19 PMC6550369 31164452

